# Efficacy and suitability of adding short-term psychodynamic psychotherapy (STPP) to pharmacotherapy in patients with depressive disorders: a systematic review

**DOI:** 10.47626/2237-6089-2023-0764

**Published:** 2025-04-17

**Authors:** Gabriele Di Salvo, Camilla Perotti, Valerio Ricci, Giuseppe Maina, Gianluca Rosso

**Affiliations:** 1 University of Torino Department of Neurosciences "Rita Levi Montalcini," Turin Italy Department of Neurosciences "Rita Levi Montalcini," University of Torino, Turin, Italy.; 2 San Luigi Gonzaga University Hospital Psychiatric Unit Turin Italy Psychiatric Unit, San Luigi Gonzaga University Hospital, Orbassano, Turin, Italy.

**Keywords:** Psychotherapy, short-term psychodynamic psychotherapy, pharmacotherapy, antidepressants, depressive disorders

## Abstract

**Objective:**

Recent guidelines on depressive disorders suggest a combination of antidepressants and psychotherapy in case of moderate to severe symptomatology. While cognitive behavioral therapy (CBT) and interpersonal therapy (ITP) are the most investigated interventions, psychodynamic psychotherapies have been less explored. The aim of this paper is to systematically review literature data on the efficacy of short-term psychodynamic psychotherapy (STPP) in combination with antidepressants in the treatment of depressive disorders, focusing on both short and long-term results and on potential moderators that could influence its effectiveness.

**Methods:**

This systematic review was conducted using the Preferred Reporting Items for Systematic Reviews and Meta-Analyses (PRISMA) guidelines. Databases searched were PubMed, Ovid, Scopus, and Cochrane Library, from inception to August 2023.

**Results:**

Adding STPP to medications in the first 6 months of treatment did not influence remission rates, but did improve acceptability, work adjustment, interpersonal relationships, social role functioning, hospitalization rates, and cost-effectiveness. After 12 months, a significant difference in remission rates arose, favoring combined therapy. From a long-term perspective, adding STPP to pharmacotherapy reduced the recurrence rate by almost 50%. STPP has proven to be more effective in longer depressive episodes, in more severe depression, and in patients with a history of childhood abuse. However, STPP had no impact on major depressive disorder (MDD) with comorbid obsessive-compulsive disorder (OCD).

**Conclusions:**

Combining STPP with antidepressants appeared to be helpful from both short-term and long-term perspectives. Still, there are few rigorous studies with large samples and further research is needed to identify which subgroups of patients may benefit more from STPP.

## Introduction

With the term short-term psychodynamic psychotherapy (STPP) we refer to a variety of psychotherapeutic techniques rooted in psychoanalysis and relying on the principles of psychoanalytic theory.^1^ Over time, these techniques developed more current and specific methodological approaches, embodying the need to evolve from traditional models.^2^ Ferenczi and Rank^3^ first postulated the need to reduce the number of sessions and the overall duration of psychotherapies in 1924, challenging the relevance of elaboration of infantile neurosis and the consequent development of personality as essential issues for therapeutic change. Later, in 1946, Alexander and French^4^ questioned the belief that short-term therapies could not lead to lasting transformation, emphasizing that recovery takes place outside the therapy sessions rather than during them. In the second half of the 20th century, Luborsky^5^ played an important role in moving beyond traditional psychoanalysis, focusing on the relevance of awareness of recurring intrapsychic and interpersonal conflicts, rather than more typical psychoanalytic issues.

This problem-centered approach proved to be highly suitable in settings with limited resources, such as public health care systems. In this regard, beyond the ideological and technical motivations that led to the development of different techniques, the need to adjust psychotherapy for public health care and hospitals was a pivotal issue during those years.^6,7^ Applicability to the system became vital, cost containment was mandatory and the lack of valuable studies documenting efficacy, appropriateness, safety, and cost-effectiveness of therapies had to be overcome.^8^ Indeed, STPP is already provided in several countries in Europe and America, such as the Netherlands, Germany, Italy, Sweden, the United Kingdom, the United States, and Mexico.^9^ In the last few decades, systematic research has been conducted into psychotherapy. Several studies have evaluated the efficacy of STPP in treating psychiatric conditions including depressive disorders.^10-16^ Notably, the need for more suitable interventions emerged when the first studies showed high rates of resistance and relapse in depressed patients treated with medications only. This need was also underscored by reinterpretation of depression not as a single entity, but as a spectrum of disorders, the clinical presentation of which is influenced by multiple variables such as emotional, cognitive, psychomotor, somatic, and personality aspects.^17^ The STPP approach aims to substantially modify the substrates of depressive disorder. This is achieved by reducing symptom severity through the expression of suppressed negative feelings, weakening feelings of guilt and inappropriateness, reinforcing self-esteem, and increasing awareness of the patient's relationships.^18^

In the nineties, some open-label uncontrolled studies underlined the capability of STPP to improve depressive symptoms in patients with depressive disorders.^10,11,19-22^ In 2003, Hilsenroth et al.^23^ confirmed and extended these findings with a more rigorous study, in which 21 subjects with major depressive disorder (MDD) underwent a 30-meeting cycle of STPP: a significant improvement in depressive symptoms and interpersonal, social, and occupational functioning, measured on both semistructured clinical interviews and self-administered questionnaires, was detected in 80% of those who completed the study. In the same decade, a meta-analysis with 416 patients highlighted an effective response at post-treatment in 45-70% of the patients with MDD treated with STPP, with a stable improvement during follow-up in 26-83% of them.^24^

More recently, a few studies were performed in order to examine whether STPP in monotherapy was as effective as pharmacotherapy for depressed patients. In 2008, STPP was compared with fluoxetine in 55 individuals with mild or moderate depression by Salminen et al.^25^ These two treatments appeared to be comparable in terms of effectiveness. This finding was later confirmed by two other studies that compared STPP to several antidepressants.^26,27^ A naturalistic 5-year follow-up study found that depressed patients treated with brief psychodynamic psychotherapy only had significantly lower recurrence rates than those treated with medications (28.3 versus 53.2%).^28^

In a recent review, our research group focused on the efficacy of STPP in monotherapy in major depression, reporting that the effectiveness of STPP is more apparent at long-term follow-up than in the immediate post-treatment period.^29^

Comparing STPP to other psychotherapeutic interventions, our research group underlined that STPP in monotherapy appeared to be more effective than brief supportive psychotherapy (BSP) in patients with moderate depression at 6 months of follow-up, while there were no significant differences in any efficacy measures between STPP and BSP in patients with mild depression.^30^ These results suggest that nonspecific interventions may be adequate to achieve improvement in patients with less severe symptoms, but that specific techniques may be essential for those with more severe forms of depression. Cognitive behavioral therapy (CBT) and interpersonal therapy (ITP) are the most investigated interventions and are the first-line recommendations for depressive disorders in the most recent guidelines,^31-34^ while psychodynamic psychotherapies have been less explored. Nevertheless, available data do not show significant differences between CBT and STPP in terms of efficacy for depressive symptoms, anxiety, pain, or quality of life,^35^ confirming the results obtained earlier by Leichsenring.^24^ Recently, a meta-analysis was performed of the efficacy of STPP in depressive disorders (both alone and in combination with antidepressants): STPP appeared to be more effective than support psychotherapy but slightly less effective than CBT.^36^

Currently, guidelines on treatment of depressive disorders recommend psychotherapy in monotherapy as the first line in cases with mild to moderate depressive symptoms, while a combination of antidepressants and psychotherapy is suggested in cases with moderate to severe symptomatology.^31,34^ However, valid integration of psychotherapy and medications still represents a significant challenge in the treatment of mental illness.

The purpose of the present paper is to systematically review the current knowledge on the efficacy of STPP in combination with antidepressants in the treatment of depressive disorders. We specifically identified two key areas that can result in more accurate assessment of outcomes as well as more effective use of STPP in addition to pharmacotherapy in depressive disorders:

1. Effectiveness (short-term and long-term results) – STPP showed different effectiveness in depressive disorders depending on the moment when it was sought. We therefore differentiated between short-term and long-term results, focusing not only on remission rates, but also on other pivotal outcomes such as treatment adherence, cost-effectiveness, occupational functioning, quality of life, hospitalization, and recurrence rates.
2. Moderators of effectiveness – STPP may be more effective in a specific type of patient. Currently, only a few studies have examined which patients could benefit specifically from STPP for depression.^9,37^ So, the question to be raised is whether it could be possible to identify smaller subgroups of patients that might benefit more from STPP than other treatments, focusing on sociodemographic characteristics, clinical features, and psychiatric comorbidities (so-called moderators).

## Methods

This systematic review was conducted using the Preferred Reporting Items for Systematic Reviews and Meta-Analyses (PRISMA) guidelines.^38,39^

Studies found were retained if they met the following criteria: (a) participants diagnosed with unipolar depressive spectrum disorders; (b) participants treated with STPP and pharmacotherapy; (c) outcome clearly defined in terms of STPP efficacy.

The evaluation was conducted by searching in different databases (PubMed, Ovid, Scopus, and Cochrane Library) from inception to August 2023. The search terms "short-term dynamic psychotherapy" and "STPP" were combined, using the Boolean operator AND, with "depressive spectrum disorders," "unipolar depression," "major depressive disorder" and "MDD." Moreover, a manual search was also conducted for possibly eligible articles from papers previously selected or from other reviews/metanalyses on the topic. We limited our research to English-language reports.

A three-step evaluation process was conducted by three authors (GDS, CP, and VR): title, abstract, and full text. The studies included were independently chosen by each author, according to inclusion criteria and clinical significance. The senior reviewers (GM and GR) were consulted in case of disagreement between authors.

## Results

**Figure 1:**
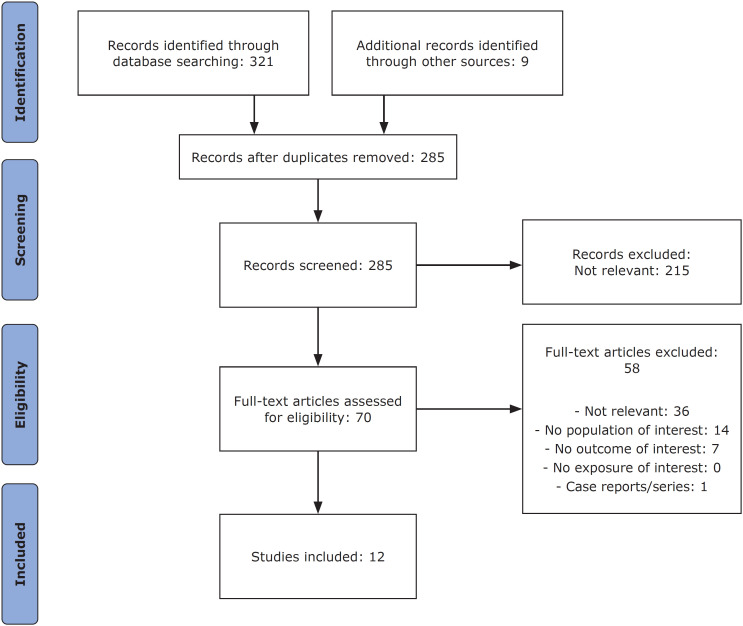
Flow diagram of the review.

A flowchart illustrating selection and inclusion of studies in this systematic review is provided in Figure 1.

A total of 285 records were identified after excluding duplicates. Seventy articles met the eligibility criteria, with 58 of them being excluded due to irrelevance, being case reports, or lacking the relevant population/outcome of interest. Ultimately, 12 papers were included. The main data of the studies included are summarized in Tables 1 and 2.

**Table 1:** Randomized controlled trials included

Title	Authors	Sample (n)	Diagnosis	Trial design	Aim
Brief dynamic therapy combined with pharmacotherapy in the treatment of major depressive disorder: long-term results	Maina et al.^40^	92	MDD, single episode	6-month acute phase (STPP + AD vs. AD) 6-month continuation phase (AD) 48-month naturalistic follow up	To evaluate recurrence rates in MDD patients responsive to acute phase combined treatment with STPP plus pharmacotherapy in comparison with patients initially treated with pharmacotherapy alone
					
Combined brief dynamic therapy and pharmacotherapy in the treatment of major depressive disorder: a pilot study	Maina et al.^41^	35	MDD	6-month acute phase (STPP + AD vs. BSP + AD) 6-month continuation phase (AD)	To compare the efficacy in MDD of STPP added to medication with that of BSP added to medication
					
Combining psychotherapy and antidepressants in the treatment of depression	de Jonghe et al.^42^	167	MDD	6 months (STPP + AD vs. AD)	To compare the efficacy of antidepressants with that of antidepressants plus STPP in MDD
					
Psychodynamic psychotherapy and clomipramine in the treatment of major depression	Burnand et al.^43^	74	MDD (also TRD)	2.5 months (STPP + AD vs. BSP + AD)	To compare a combination of clomipramine and STPP with clomipramine alone in MDD
					
Evaluation of an outpatient intervention for women with severe depression and a history of childhood trauma	Vitriol et al.^44^	87 women	MDD, severe episode, with childhood trauma	3 months (STPP + AD vs. BSP + AD)	To assess the effectiveness of a structured intervention developed for women with severe depression and childhood trauma by comparing it to standard treatment
					
Differential efficacy of cognitive behavioral therapy and psychodynamic therapy for major depression: a study of prescriptive factors.	Driessen et al.^45^	233	MDD	5.5 months (STPP/CBT, both with and without AD)	To identify patient characteristics that might moderate differential treatment effects between STPP + antidepressants vs. CBT + antidepressants
					
No effect of adding brief dynamic therapy to pharmacotherapy in the treatment of obsessive compulsive disorder with concurrent major depression	Maina et al.^46^	57	OCD with concurrent MDD (also TRD)	4-month acute phase (STPP + AD vs. AD) 8-month continuation phase (AD)	To explore the efficacy of STPP combined with pharmacotherapy in comparison with pharmacotherapy alone in the treatment of OCD with concurrent MDD
					
Brief dynamic therapy combined with pharmacotherapy in the treatment of panic disorder with concurrent depressive symptoms	Martini et al.^47^	39	Panic disorder with concurrent depressive symptoms	4-month acute phase (STPP + AD vs. BSP + AD) 8-month continuation phase (AD)	To compare the efficacy of STPP and brief supportive therapy in the combined treatment of panic disorder with concurrent depressive symptoms

AD = antidepressants; BSP = brief supportive psychotherapy; CBT = cognitive-behavioral therapy; MDD = major depressive disorder; OCD = obsessive-compulsive disorder; STPP = short-term psychodynamic psychotherapy; TRD = treatment-resistant depression.

**Table 2:** Systematic reviews and meta-analyses included

Title	Authors	Studies included (n)	Diagnosis	Topic	Aim
The efficacy of adding short-term psychodynamic psychotherapy to antidepressants in the treatment of depression: a systematic review and meta-analysis of individual participant data	Driessen et al.^48^	7 studies (482 patients)	MDD or another unipolar mood disorder	STPP + AD	To examine the efficacy of adding STPP to antidepressants in the treatment of depression
					
Which patients benefit from adding short-term psychodynamic psychotherapy to antidepressants in the treatment of depression? A systematic review and meta-analysis of individual participant data	Driessen et al.^37^	7 studies (482 patients)	MDD or another unipolar mood disorder	STPP + AD	To examine efficacy moderators of combined treatment (STPP + antidepressants) vs. antidepressants for adults with depression
					
Psychological therapies for treatment-resistant depression in adults	Ijaz et al.^49^	6 studies	Treatment-resistant depression	Different types of psychotherapy plus AD	To assess the effectiveness of adding psychotherapies to AD for adults with treatment-resistant depression
					
Efficacy of short-term psychodynamic psychotherapy (STPP) in depressive disorders: A systematic review and meta-analysis.	Caselli et al.^36^	31 studies	MDD or another unipolar mood disorder	STPP, both with and without AD	To evaluate the efficacy of STPP in depression by comparing STPP with different types of interventions

AD = antidepressants; MDD: major depressive disorder; STPP = short-term psychodynamic psychotherapy.

All studies considered the addition of STPP to antidepressants in patients with depressive disorders *ab initio*; none of the studies in the literature analyzed the potential efficacy of sequential combination strategies.

### Effectiveness

#### Short-term results

From a short-term perspective, the literature indicates that there is no significant difference in remission rates after 6 months of treatment between depressed patients treated with antidepressants alone and those who received a combination of antidepressants and STPP. In a study conducted by Maina et al.^40^ with 92 patients with MDD, 64.1% achieved remission with combined treatment, while 61.4% achieved remission with medication monotherapy.

Our research group reported another interesting finding: after 6 months of combined treatment, during the continuation phase (when only medication is provided), a substantial number of patients who had been treated with STPP achieved remission or showed a further clinical improvement, while some patients treated only with antidepressants lost their positive results. In a randomized clinical trial (RCT), STPP was compared to BSP (both combined with antidepressants) in 35 patients with MDD: after 6 months of the continuation phase (so 1 year after the beginning of the treatment), not only did a significant reduction in symptomatology emerge on the Hamilton Depression Rating Scale (HAMD) and on the Clinical Global Impression (CGI) total scores (favoring combined therapy), but statistically significant intergroup differences also appeared in terms of remission rates.^41^

This evidence was recently confirmed by a large meta-analysis run by Driessen et al.^48^: a 1.5 HAMD point difference, with no significant effect size, was highlighted at post-treatment between the patients treated with combined therapy and those treated with medication only. This difference in HAMD increased up to 2.9 points (with a significant effect size) after another 6 months/1 year of follow-up.

Aside from remission rates and improvement in depressive symptoms, there are other pivotal outcomes to be considered at this stage.

In this regard, in their RCT with 167 patients with MDD, De Jonghe et al.^42^ showed combined therapy was more acceptable than pharmacotherapy alone (13 versus 32% of patients, respectively, refused the treatment), with a significant difference in pharmacotherapy dropout rates between the two groups: 40 versus 22% in favor of combined therapy.

In the same period, the cost-effectiveness of adding STPP to medication in depressed patients was evaluated in an RCT with 74 MDD patients treated with clomipramine alone or with clomipramine plus STPP. Different outcomes such as treatment effect, work effectiveness, hospitalization rates, and costs, both at 10 weeks of treatment and at discharge were analyzed. While no treatment effect was found at 10 weeks (in accordance with the results published by Maina^40^ and Driessen^48^), adjustment to work, measured with the subscale of the modified Health-Sickness Rating Scale (HSRS), was found to be significantly better in the combined group at 10 weeks of treatment. Moreover, assignment to combined treatment was associated with fewer working days lost (34.5 ± 23.0 days compared with 56.2 ± 34.6 days) and with a lower rate and fewer days of hospitalization. Combined treatment was also associated with an overall saving – including direct and indirect costs – of $ 2,311 per patient ($ 3,394 among those who were in stable employment when they entered the study).^43^

Referring to quality of life, Vitriol et al.^44^ examined the effectiveness of a 3-month structured outpatient intervention developed for 87 women with severe depression and childhood trauma. Better interpersonal relationships and social role functioning (measured with Lambert's Outcome Questionnaire [OQ-45.2]) after 6 months were observed in the 44 patients treated with antidepressants plus STPP, compared with the 43 patients treated with antidepressants only.

#### Long-term results

To date, only one trial has assessed the long-term efficacy of adding STPP to pharmacotherapy. Maina et al.^40^ evaluated recurrence rates in 92 unipolar major depression patients who were responsive to acute phase combined treatment (STPP plus pharmacotherapy), in comparison with patients initially treated with pharmacotherapy alone. This naturalistic study included a 4-year follow-up (without any treatment) and showed that patients who had received combined treatment had a significantly lower rate of recurrences of depressive episodes at the end of the follow-up period (27.5 versus 46.9%).

### Moderators of effectiveness

#### Socio-demographic characteristics

Driessen et al.^37^ have recently run a meta-analysis specifically focused on features that may influence the response to adding STPP to antidepressants in 482 subjects with depressive disorders. They showed better efficacy at post-treatment in participants with ≤ 8 years in education rather than 13-15, but this interaction appeared to be no longer statistically significant when only low risk of bias studies were considered.

We could not find other evidence about how socio-demographic characteristics may influence the response to STPP in depressive disorders.

#### Clinical features

Concerning the clinical features that could influence the effectiveness of adding STPP to antidepressants in depressive disorders, we found some evidence about characteristics such as severity, anxiety levels, and duration of the current depressive episode. We also addressed whether adding STPP to antidepressants could be an option for treatment-resistant depression (TRD).

In a recent, already-mentioned, meta-analysis of individual participant data of 482 depressed patients, the effectiveness of adding STPP to medications appeared to be greater in patients with higher HAMD scores than in those with lower symptom severity, both at post-treatment and in follow-up. These results confirmed severity as a moderator of STPP effectiveness.^37^

Focusing on other clinical moderators associated with differential efficacy of STPP and CBT in MDD, in an RCT with 233 adults with major depressive episode, STPP proved to be more efficacious than CBT among patients with low anxiety levels. However, this result was only observed in moderately depressed patients treated with psychotherapy only; the authors assumed this evidence could be explained by the fact that patients with less anxiety may be better able to enter into the insight-oriented dialog of STPP.^45^ Nevertheless, a meta-analysis run by the same group highlighted different results in patients treated with both STPP and antidepressants: at follow-up, the effectiveness of combined therapy was greater in those with higher baseline anxiety symptom levels, even if this difference was no longer statistically significant when modeling all significant moderators simultaneously.^37^

The abovementioned 2016 study by Driessen et al.^45^ drew attention to another moderator: the duration of the current depressive episode, which is linked to the varying effectiveness of STPP in high-severity patients receiving both psychotherapy and medications. CBT appeared to be more efficacious than STPP for patients with current episode duration < 1 year, while STPP was more efficacious than CBT in those with current episode duration ≥ 1 year. The research team hypothesized that the difference observed might be attributable to the stronger impact of personality structure on symptoms in patients with longer depressive episodes. This could lead to development of more intricate working alliances and transference feelings, which can be effectively utilized in STPP rather than in CBT. This evidence was later confirmed in a meta-analysis: efficacy was greater at post-treatment for patients with a depressive episode duration of > 2 years than in either those with < 1 or 1-2 years’ duration.^37^

Only two studies assessing the efficacy of STPP in depression included patients with TRD in the sample.^43,46^ So, the question to be raised is whether the efficacy of STPP is different in those who failed first-line treatments. In this regard, adding psychotherapy to antidepressants did appear to be beneficial for depressive symptoms and for remission rates for patients with TRD.^49^ However, this study included trials with CBT, intensive short-term dynamic psychotherapy, ITP, and group dialectical behavioral therapy, so data specific to STPP are lacking.

#### Comorbidities

Concerning how comorbidities could influence the effectiveness of adding STPP to antidepressants in depressive disorders, we found studies focused on obsessive-compulsive disorder (OCD), panic disorder, and childhood abuse.

Regarding OCD, a number of investigations have reported that patients with OCD and comorbid depression are less responsive to both pharmacotherapy and cognitive-behavioral techniques. Thus, these patients need to be targeted as a special population for treatment studies. A study conducted on OCD patients by our research group did not identify a significant impact of adding STPP to antidepressants in treating either depressive or obsessive symptoms; it is noteworthy how a comorbid condition can considerably influence the treatment outcome, given the efficacy of STPP in treating depression *per se*.^46^ Thus, new treatment options still need to be explored for these patients.

Few studies addressed the question of the clinical utility and efficacy of the combination of STPP and pharmacotherapy in the treatment of panic disorder with concurrent depression. An RCT with 35 patients with panic disorder and depressive symptoms found that adding STPP is effective and preferable to supportive psychotherapy in these patients.^47^ However, the meta-analysis by Driessen et al.^37^ found STPP plus medications was more effective at follow-up in patients without anxiety disorder comorbidity, although this effect was no longer statistically significant when studies that only included participants with specific comorbidities were excluded.

Focusing on childhood abuse, in an already-mentioned study, Vitriol et al.^44^ explored the efficacy of STPP in 87 women with severe depression and a history of childhood trauma. STPP appeared to be more effective than standard treatment in improving depressive symptoms, interpersonal relationships, and social role functioning. Therefore, screening for past traumas could be useful to steer the patient to a more specific intervention.

## Discussion

The aim of this paper was to review literature data on the efficacy of STPP in combination with antidepressants in the treatment of depressive disorders. In particular, compared to systematic reviews conducted on the subject in recent years,^37,48^ our study also takes into consideration the effects of adding STPP on other short-term outcomes (acceptability, work adjustment, interpersonal relationships, social role functioning, rates of hospitalization, and costs of intervention), in TRD, and on the prevention of long-term recurrences.

Although adding STPP to antidepressants is widely considered an effective therapeutic option for patients with depressive disorders and showed efficacy comparable with CBT and IPT, the empirical support for this statement is still quite small. Most of the available data comes from studies with small sample sizes, lacking statistical power. This may explain why STPP ranks second to CBT and IPT. Recently, a number of large-scale and high-quality studies have been conducted and others are being carried out.

Analyzing the available data, despite the above-mentioned limitations, combining STPP with antidepressants appeared to be helpful from both a short-term and a long-term perspective. The size of this effect was small at post-treatment and moderate at follow-up. In particular, adding STPP in the first 6 months of treatment did not appear to influence remission rates, but did improve acceptability (reducing drop-out rates), work adjustment, interpersonal relationships, social role functioning, and rates of hospitalization, and also allowed consistent overall savings. At the end of the continuation phase (12 months), a significant difference emerged in remission rates in patients treated with antidepressants plus STPP versus those treated with just medication. From a long-term perspective (4 years of naturalistic follow-up), adding STPP to pharmacotherapy reduced recurrence rates by almost 50%.

Moving towards a more tailored treatment, we identified some features that appeared to influence the response: STPP has proven to be more effective in longer depressive episodes, in more severe depressions, and in patients with a history of childhood abuse. However, STPP showed no impact on MDD with comorbid OCD. Focusing on concomitant anxiety levels/comorbidity, we found contrasting results, so further studies are needed. Basically, it remains largely unclear which patients can benefit specifically from STPP for depressive disorders, because relatively few studies have been conducted and the sample sizes are often small.

The results of the review, pertaining both to the efficacy of adding STPP to antidepressants and to potential moderators of effectiveness, should be considered in the light of its primary limitation, which is the scarcity of rigorous studies and thus the low number of includable papers.

In conclusion, adding STPP to medications can potentially become a major asset in the treatment of depressive disorders. The key strengths of STPP lie in its effectiveness in preventing depressive recurrences and its potentially high applicability to public health services due to its problem-centered approach and consequent shorter duration compared to other psychodynamic psychotherapeutic techniques. One potential obstacle to the dissemination of STPP throughout routine clinical practice is the limited availability of adequately trained therapists: this limitation is attributable to the scarcity of schools providing specialized training and supervision as well as to the general shortage of financial resources and personnel in the public healthcare system. This review highlights that STPP is a technique not suitable for all patients with depressive disorders, but rather one that is preferable for those with specific characteristics. Still, there are few rigorous studies with large samples and further research is needed to identify which subgroups of patients can benefit more from STPP.
